# Supplementary material for: Association-induced folding governs surrogate light chain and pre-B cell receptor core assembly
Source: Nat Commun. 2026 Jan 30;17:1202. doi: 10.1038/s41467-026-68965-5 (PMC12859133; doi:10.1038/s41467-026-68965-5)
Supplement: Supplementary file 1 — Supplementary Information [file 41467_2026_68965_MOESM1_ESM.pdf]

Association-induced folding governs surrogate light chain and B cell receptor core assembly

Supplementary Information

Jasmin König[1,2], Natalia Catalina Sarmiento Alam[1,2], Ruiming He[1,2], Nicolas Blömeke[1,2], Olga Sieluzycka[1], Florian Rührnößl[1,2], Maximilian Riedl[1,2], Bernd Reif[1,3], Matthias J. Feige[1,2] and Johannes Buchner[1, 2]*

[1] Department Bioscience, School of Natural Sciences, Technical University Munich, Garching, Germany

[2] Center for Functional Protein Assemblies, Technical University Munich, Ernst-Otto-Fischer-Strasse 8, 85748 Garching, Germany

[3] Institute of Structural Biology (STB), Helmholtz-Zentrum München (HMGU), Ingolstädter Landstr. 1, 85764 Neuherberg, Germany

* to whom correspondence should be addressed: johannes.buchner@tum.de

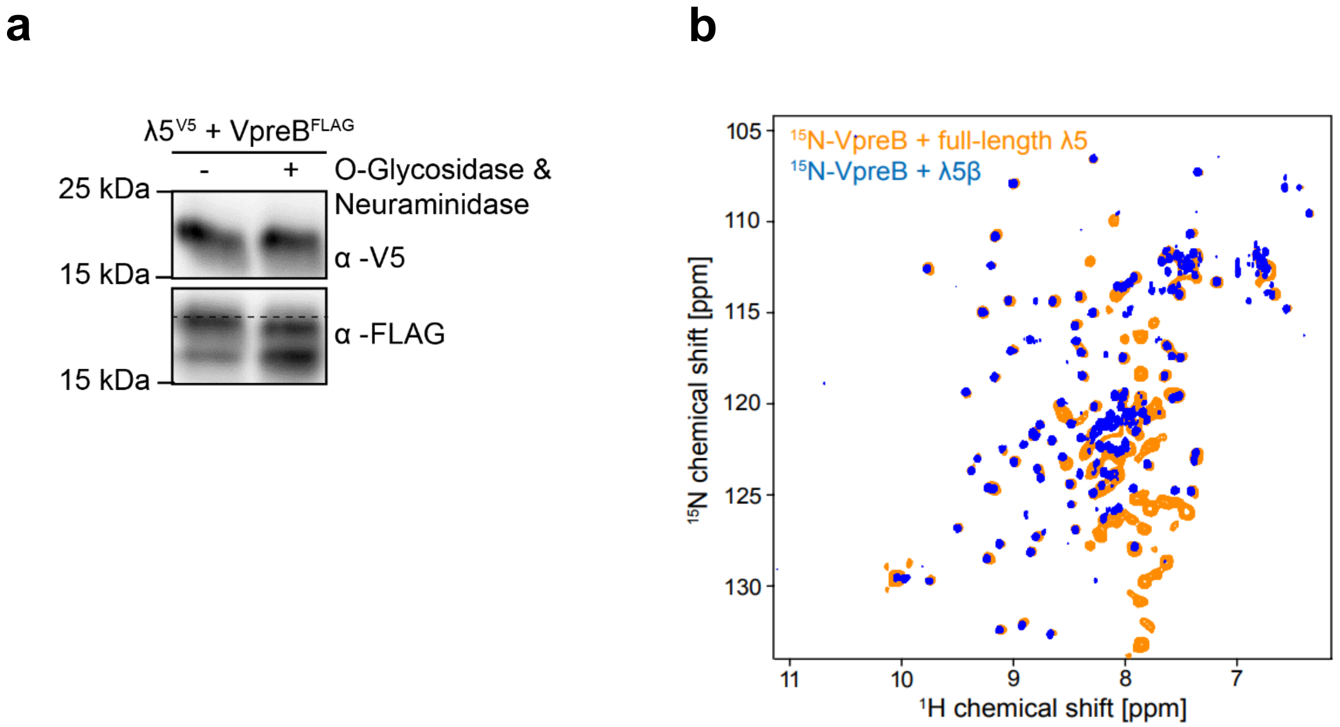

Supplementary Fig. 1 a Glycosylation test for Λ5 and VpreB. During secretion, VpreB becomes O-glycosylated, showing that the SLC traverses the Golgi apparatus. This figure shows full, uncropped scans corresponding to the blots presented in Supplementary Fig. 6. The experiment was performed as triplicate. **b** ^{1}H,^{15}N correlation spectra of 100 µM VpreB purified as a complex with unlabelled λ5 C212S (orange), superimposed with a spectrum obtained from a 100 µM VpreB sample that was incubated with a 200 µM solution of unlabelled λ5β peptide (blue). The complex of VpreB with full length λ5-C212S is not stable for prolonged NMR measurements. This is apparent from the strong cross peaks which appear at proton chemical shifts around 7.8 ppm. All experiments were recorded at 600 MHz, setting the sample temperature to 25 °C.

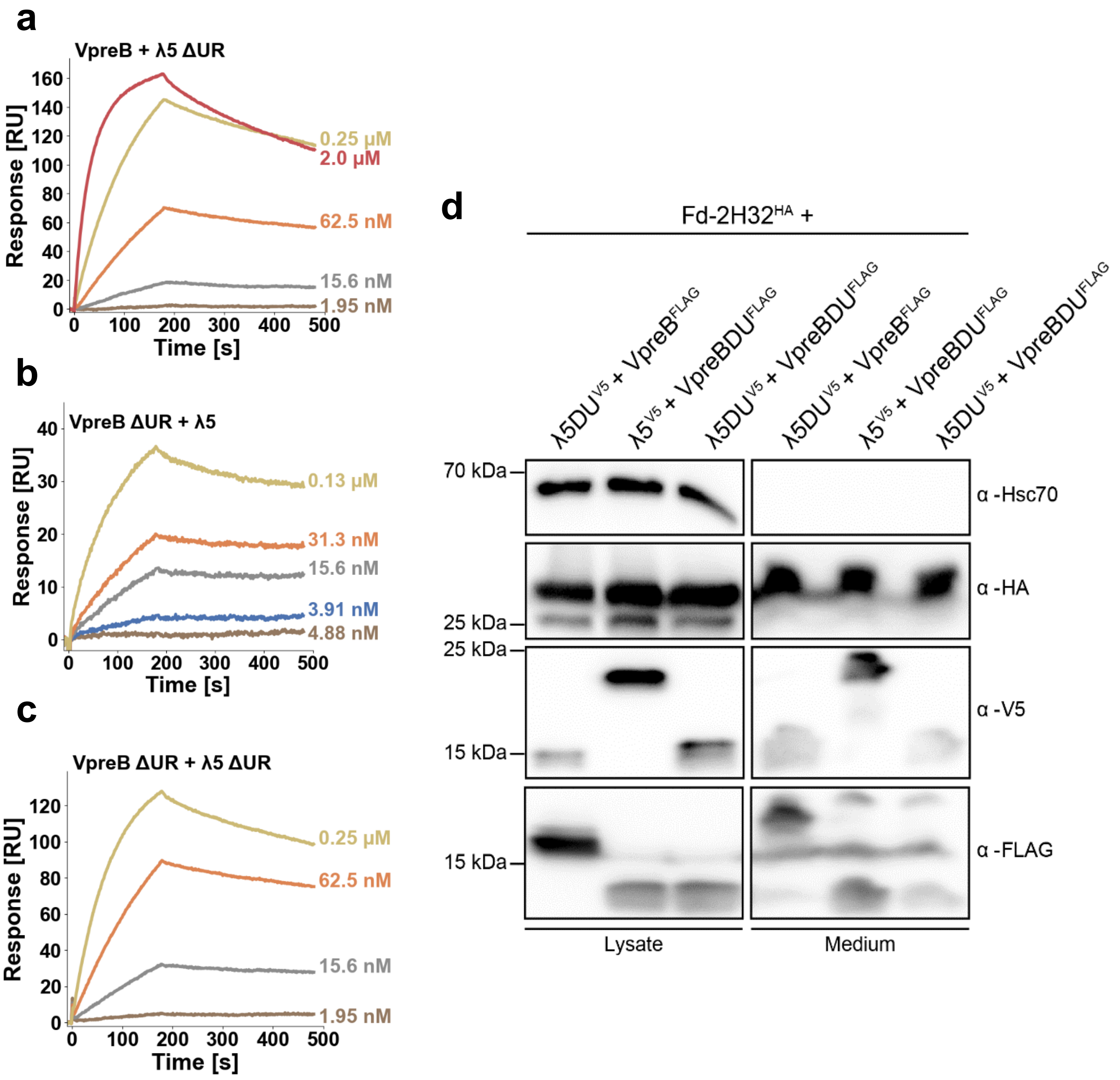

Supplementary Fig. 2 Role of the unique regions in the SLC. The dissociation constant (K_D) and association kinetics constants (k_{on}, k_{off}) were determined for λ5 ΔUR and VpreB (**a**), λ5 and VpreB ΔUR (**b**), λ5 ΔUR and VpreB ΔUR (**c**). The experiment was performed as duplicate (n=2). **d** Expression and secretion of human Fd-2H32 with ΔUR-variants of λ5 and VpreB in HEK293T cells. This figure shows full, uncropped scans corresponding to the blots presented in Supplementary Fig. 6. The experiment was performed as duplicate.

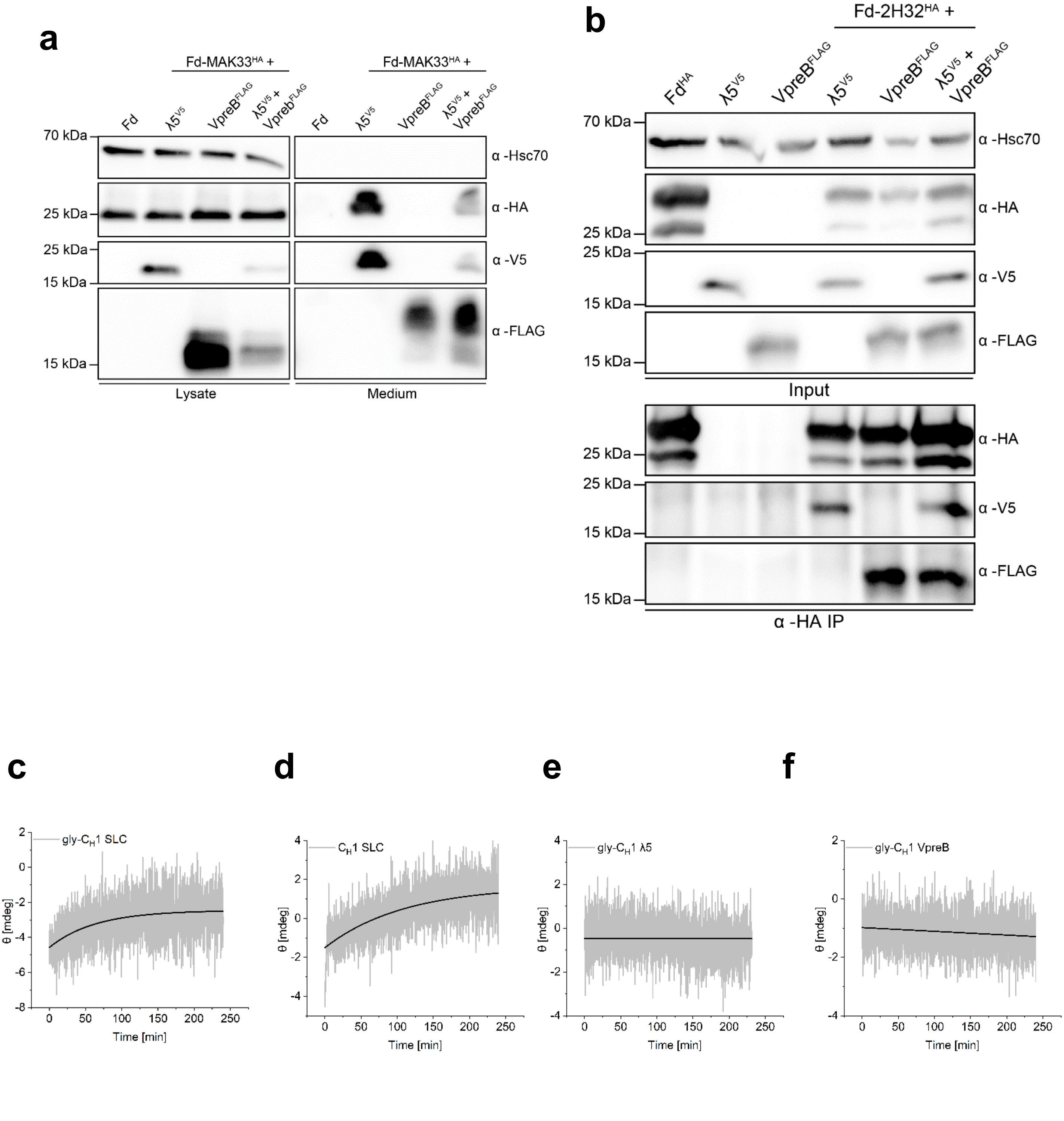

Supplementary Fig. 3 Assembly of Fd and SLC. a Expression and secretion of murine Fd-MAK33 alone and with λ5, VpreB and the SLC in HEK293T cells. The experiment was performed as duplicate (n=2). **b** Immunoprecipitation of human Fd-2H32 with λ5, VpreB and the SLC in HEK293T cells. This figure shows full, uncropped scans corresponding to the blots presented in Supplementary Fig. 6. The experiment was performed as duplicate (n=2). The folding of glycosylated C_H1 **(c)** and unglycosylated C_H1**(d)** 2H32 by SLC was monitored by the change in the CD signal at 205 nm. A single exponential trace was observed and fitted by an exponential decay function to obtain the folding rate of the reaction. The folding of glycosylated C_H1 2H32 by λ5 **(e)** and VpreB **(f)** was monitored by the change in the CD signal at 205 nm. A single exponential trace was observed and fitted by an exponential decay function to obtain the folding rate of the reaction. The experiment was performed as duplicate (n=2).

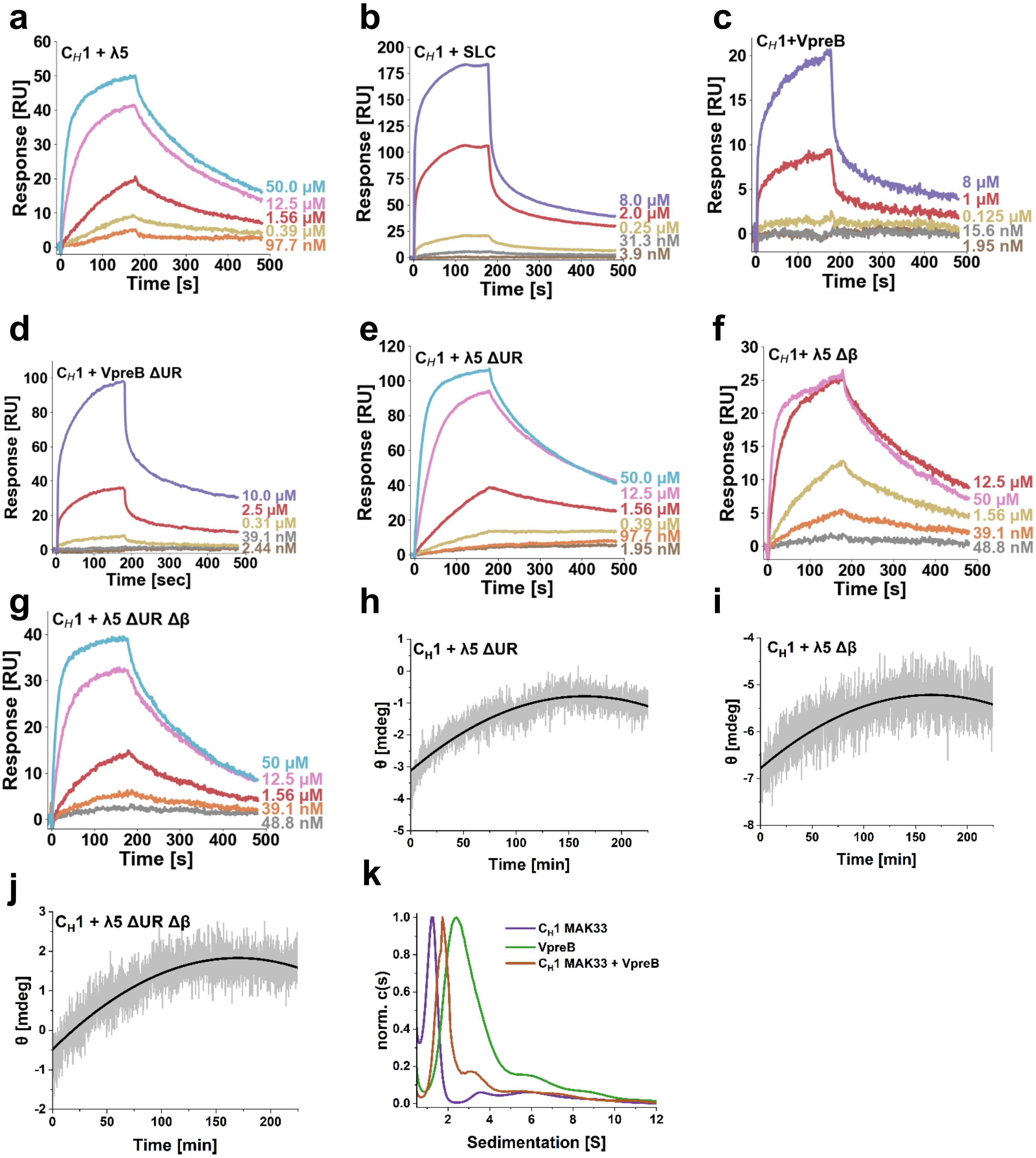

Supplementary Fig. 4 Assembly of the C_H1 MAK33 domain with the SLC and variants. The dissociation constant (K_D) and association kinetics constants (k_{on}, k_{off}) were determined for C_H1 MAK33 with Λ5 (**a**), SLC (**b**), VpreB (**c**), VpreB ΔUR (**d**), λ5 ΔUR (**e**), λ5 Δβ (**f**), λ5 ΔUR Δβ (**g**) by SPR experiments in duplicates. The folding of C_H1 MAK33 by λ5 ΔUR (**h**), λ5 Δβ (**i**), λ5 ΔUR Δβ (**j**) and λ5 3A (**l**) was monitored by the change in signal at 205 nm by far-UV CD spectroscopy. The experiment was performed as duplicate (n=2). A single exponential trace was observed and fitted by an exponential decay function to obtain the folding rate of the reaction. **k** The heterodimer of C_H1 and VpreB was measured by AUC. The experiment was performed once (n=1).

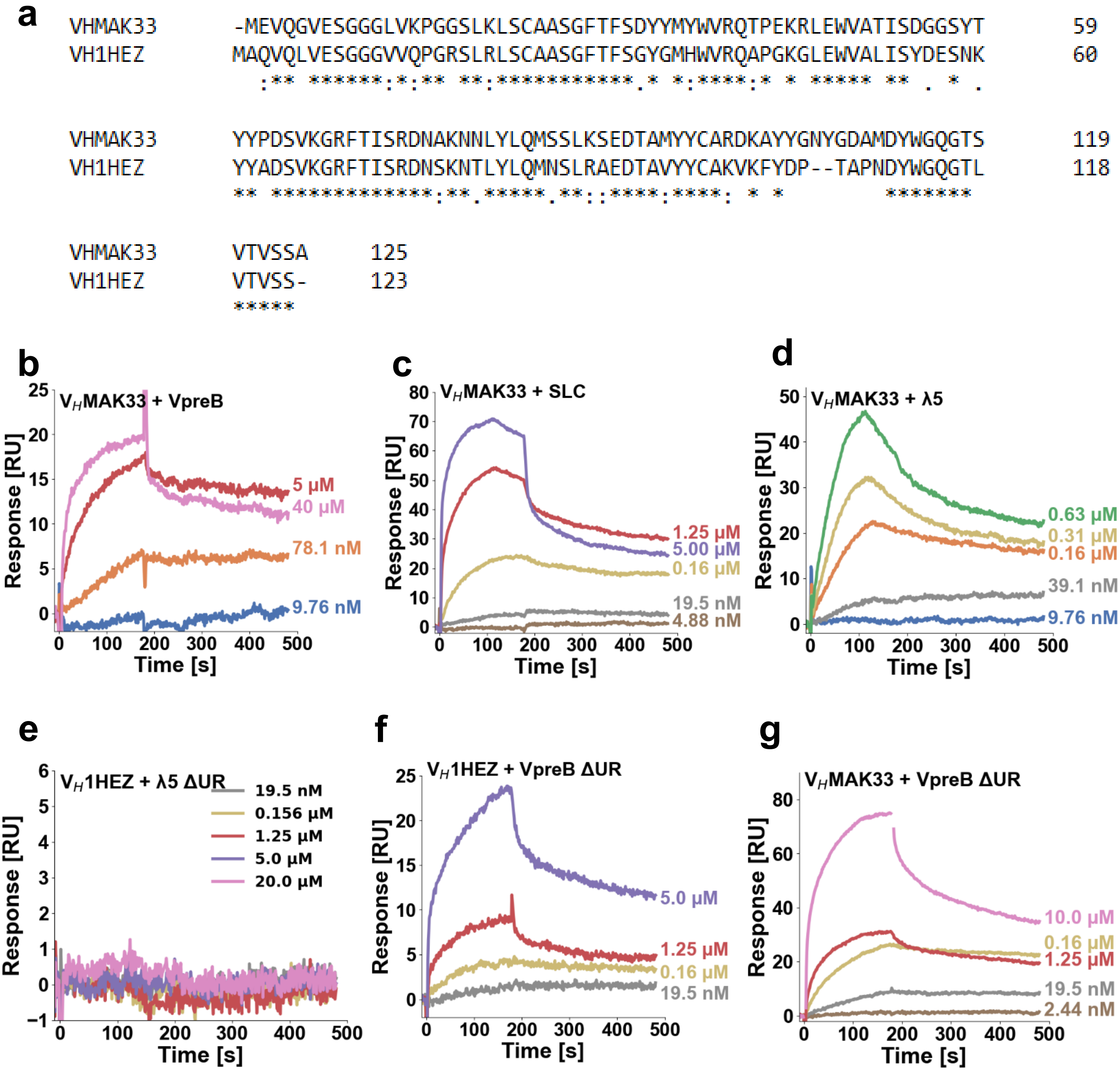

Supplementary Fig. 5 Role of the unique regions in the SLC the interaction with V_H. a Alignment of the amino acid sequence of V_H 1HEZ and V_H MAK33 with ClustalW. The sensorgrams show the interaction analysis by SPR of VpreB with V_H Mak33 (**b**), SLC with V_H Mak33 (**c**), and of λ5 and V_H Mak33 (**d**), of V_H 1HEZ with λ5 ΔUR (**e**), V_H 1HEZ with VpreB ΔUR (**f**), V_H MAK33 with VpreB ΔUR (**g**). The experiment was performed as duplicate (n=2).

Supplementary Table 1. HDX-MS summary table.

Protein	Condition	Sequence length	Time points (min)	Coverage (%)	No. of peptides	Avg peptide length	Replicates	Repeatability (avg SD)	Software
VpreB	VpreB alone	126	0, 0.17, 1, 10, 30, 120	100	74	11.18	2 independent experiments; 3 technical replicates each	0.0625	Deuteros
VpreB in SLC C215S Complex	VpreB in SLC Complex with λ5 C215S	126	0, 0.17, 1, 10, 30, 120	100	74	11.18	2 independent experiments; 3 technical replicates each	0.0413	Deuteros
λ5 C215S	λ5 C215S alone	169	0, 0.17, 1, 10, 30, 120	98.22	77	11.66	2 independent experiments; 3 technical replicates each	0.0588	Deuteros
λ5 C215S in SLC complex	λ5 C215S in SLC complex with VpreB	169	0, 0.17, 1, 10, 30, 120	98.22	77	11.66	2 independent experiments; 3 technical replicates each	0.0574	Deuteros

*SLC denotes the surrogate light chain complex composed of VpreB and λ5.

Supplementary Table 1. HDX-MS summary table for VpreB and λ5 analyzed alone or in the surrogate light chain (SLC) complex.

Supplementary Fig. 1a

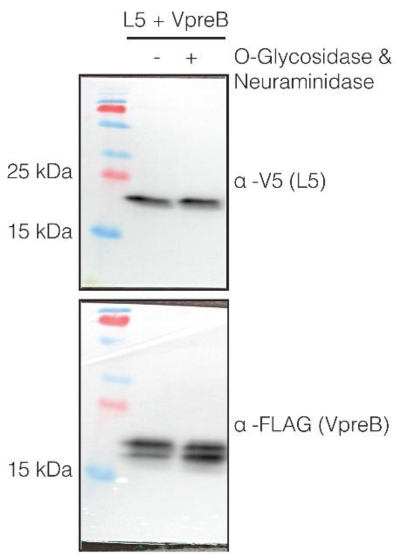

Supplementary Fig. 2d

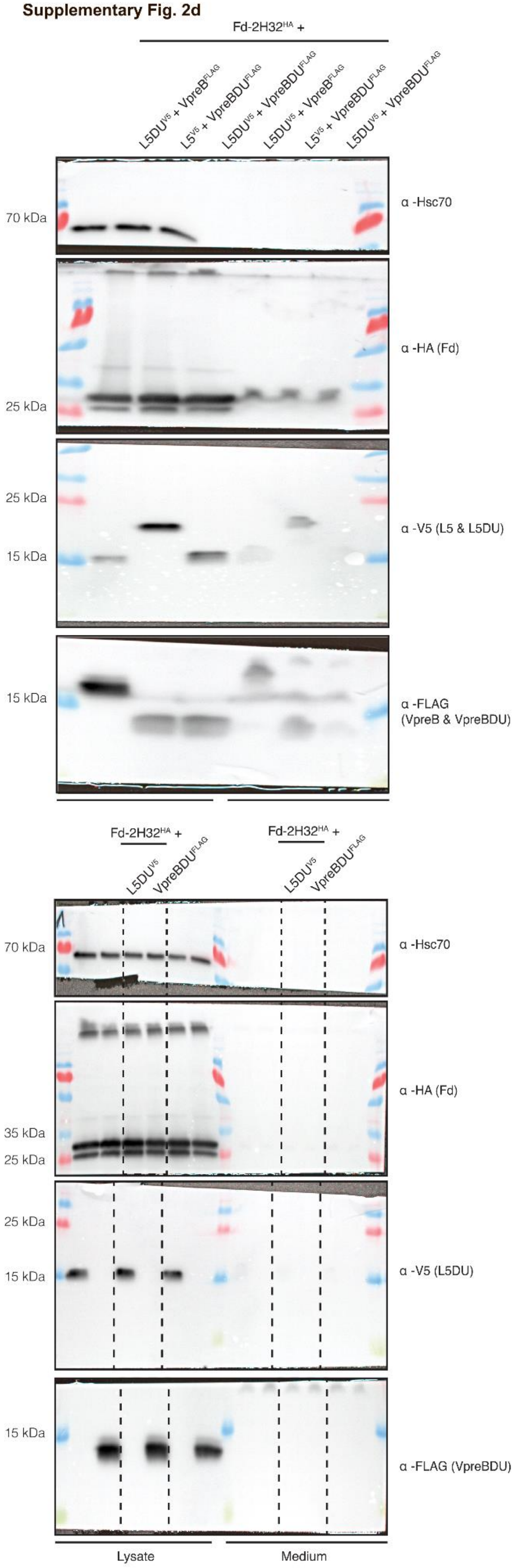

Supplementary Fig. 3a

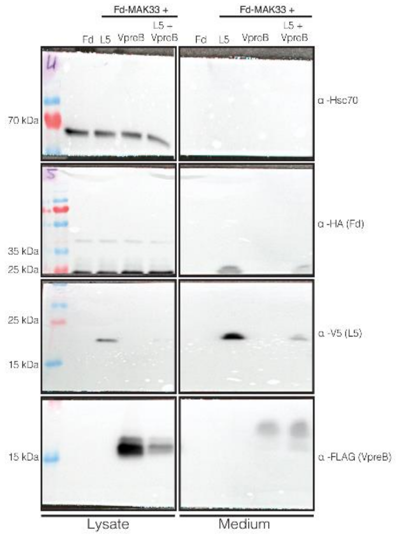

Supplementary Fig. 3b

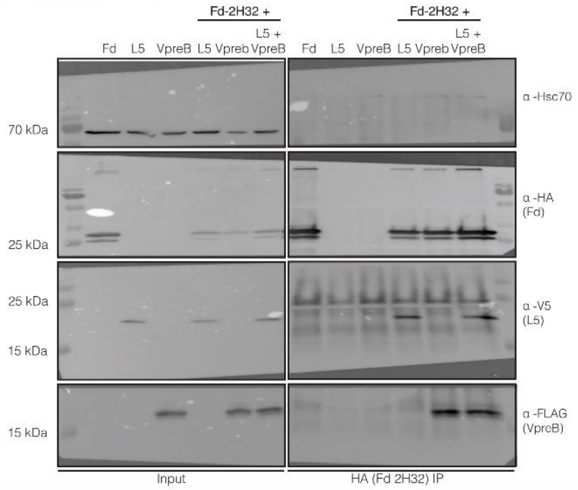

Source Data file for Supplementary Fig. 1a, 2d, 3a, and 3b: Uncropped scans of the blots corresponding to the panels shown.
